# RETRACTION: Synthesis and Optimization of MoS_2_@Fe_3_O_4_‐ICG/Pt(IV) Nanoflowers for MR/IR/PA Bioimaging and Combined PTT/PDT/Chemotherapy Triggered by 808 nm Laser

**DOI:** 10.1002/advs.75048

**Published:** 2026-04-09

**RETRACTION**: B. Liu, C. Li, G. Chen, B. Liu, X. Deng, Y. Wei, J. Xia, B. Xing, P. Ma, and J. Lin, “Synthesis and Optimization of MoS_2_@Fe_3_O_4_‐ICG/Pt(IV) Nanoflowers for MR/IR/PA Bioimaging and Combined PTT/PDT/Chemotherapy Triggered by 808 nm Laser,” *Advanced Science* 4, no. 8 (2017): 1600540. https://doi.org/10.1002/advs.201600540.

The above article, published online on 10 April 2017 in Wiley Online Library (wileyonlinelibrary.com), has been retracted by agreement between all authors; the journal Editor‐in‐Chief, Kirsten Severing; and Wiley‐VCH GmbH. An investigation by the publisher found that the article lacks appropriate declarations of approval for animal experiments by an appropriate ethics body.

Additionally, a third party reported concerns on PubPeer [1] that the tumors depicted in Figure 7D exceeded 2 cm in any one dimension, which is currently considered the maximum size/volume for humane endpoints for mice in laboratory experiments [2]. While guidelines may vary by institution and country‐specific ethics requirements, *Advanced Science* strongly recommends compliance with the US NIH's Policy on Animal Research Advisory Committee Guidelines [3] and states this clearly in the author guidelines.

Authors P. Ma and J. Lin responded to an inquiry by the publisher and reported that the original ethics approval documentation and experimental records were no longer available. Additionally, the authors reported that at the time the work was completed, their research did not follow guidelines regarding tumor volume limitation and timely euthanasia, which resulted in excessive tumor size. As such, the authors requested the retraction of their article.

The retraction has been agreed to because the study does not have evidence of ethics approval, and because the research conducted in this article did not meet ethical standards for animal experiments at the time the study was conducted.
